# Modeling NDD-Associated NLGN2 Depletion Using CRISPR/Cas13 Reveals Exaggerated Process Elongation Mediated by the CCDC88A-G Protein–ELMO Axis

**DOI:** 10.3390/ijms27177612

**Published:** 2026-08-25

**Authors:** Hideji Yako, Mikito Takahashi, Shiori Tada, Mitona Waragai, Yuki Miyamoto, Junji Yamauchi

**Affiliations:** 1 Laboratory of Molecular Neuroscience and Neurology, Tokyo University of Pharmacy and Life Sciences, 1432-1 Horinouchi, Hachioji, Tokyo 192-0392, Japan; hyako@toyaku.ac.jp (H.Y.); miyamoto-y@ncchd.go.jp (Y.M.); 2 Department of Pharmacology, National Research Institute for Child Health and Development, Setagaya, Tokyo 157-8535, Japan; 3 Department of Biological Sciences, Tokyo College of Biotechnology, Ota, Tokyo 157-8535, Japan

**Keywords:** NDD, NLGN2, CCDC88A, ELMO1, Rac1, neurite outgrowth

## Abstract

Neuroligin-2 (NLGN2) is a cell adhesion molecule implicated in neurodevelopmental disorders (NDDs), including autism spectrum disorder (ASD) and intellectual disability (ID). While NLGN2 is well known as a postsynaptic organizer of predominantly inhibitory synapses, accumulating evidence suggests that NLGN family proteins are also involved in neuronal morphogenesis during early developmental stages. However, a gap remains in our understanding of how loss of function in NLGN2 potentially leads to abnormal neuronal morphogenesis. Here, we investigated the molecular basis of excessive neuronal process formation induced by depletion of NLGN2 using the N1E-115 cell line, an established model of neuronal differentiation. Clustered regularly interspaced short palindromic repeat (CRISPR)/Cas13-mediated knockdown of NLGN2 promoted neuronal process elongation and neuronal differentiation marker expression. Mechanistically, NLGN2 knockdown resulted in activation of coiled-coil and hook domain-containing protein 88A (CCDC88A, also known as Girdin or GIV), a non-receptor guanine nucleotide exchange factor for heterotrimeric G proteins. Transfection of the regulator of G protein signaling (RGS) domain of RGS3, a negative regulator of G proteins, or the G protein-binding domain of engulfment and cell motility 1 (ELMO1) effectively decreased the excessive process elongation phenotype. Similar effects were observed in primary cortical neurons. Furthermore, these interventions normalized elevated Rac1 activity induced by NLGN2 knockdown. Collectively, our findings identify the CCDC88A-G protein-ELMO signaling pathway as a key mediator of excessive neuronal morphogenesis following NLGN2 knockdown. These results provide valuable insight into the mechanisms by which NLGN2 dysfunction may contribute to abnormal neuronal morphogenesis and suggest potential recovery strategies.

## 1. Introduction

Neurodevelopmental disorders (NDDs) include autism spectrum disorder (ASD), intellectual disability (ID), and attention-deficit/hyperactivity disorder (ADHD) [1,2,3]. These disorders are characterized by impairments in cognitive, social, and behavioral functions arising from abnormal brain development [1,2,3]. Recent genetic studies have identified numerous NDD-associated genes and their products that regulate synapse formation and neuronal connectivity [4,5,6]. In addition to defects in synaptic transmission, abnormal neuronal cell and neural tissue morphogenesis is increasingly recognized as an important pathological hallmark contributing to NDD pathogenesis [4,5,6].

Neuroligins (NLGNs) are postsynaptic cell adhesion molecules that interact with presynaptic neurexins (NRXNs) to regulate synapse formation and maturation [7,8,9,10]. Among the NLGN family members, NLGN2 is predominantly localized at inhibitory synapses and plays a critical role in the assembly and maintenance of these synaptic structures in adults [7,8]. Genetic variants, particularly loss-of-function variants, and decreased expression levels of NLGN2 have been associated with ASD, ID, anxiety-related disorders, and other neuropsychiatric conditions, highlighting its importance in normal brain development [7,8]. While NLGN2 has been studied as a regulator of synaptic organization, accumulating evidence suggests that it also participates in earlier developmental events, including morphogenesis [11,12,13,14]. However, the molecular mechanisms by which NLGN2 dysfunction affects neuronal cell morphogenesis remain to be elucidated.

Neurite extension and neuronal morphogenesis are tightly controlled by signaling pathways that regulate many types of cytoskeletal protein dynamics [15,16,17,18]. Small GTPases of the Rho family, particularly Rac1, are key regulators of cell morphogenesis [19,20]. Rac1 activity can be stimulated through multiple upstream signalosome molecules, including engulfment and cell motility (ELMO) family proteins [21,22]. ELMO family proteins function as scaffold proteins of Rac1-specific dedicator of cytokinesis (DOCK) family guanine nucleotide exchange factors (DOCK-A and DOCK-B subfamilies) [21,22]. Recent studies have suggested functional interactions between heterotrimeric G proteins and ELMO family proteins, providing a plausible molecular mechanism for regulating neuronal cell morphological changes through activation of Rac1, a master regulator of cell morphogenesis [19,20,23,24]. Notably, coiled-coil and hook domain-containing protein 88A (CCDC88A, also known as Girdin or GIV) functions as a non-receptor guanine nucleotide exchange factor for heterotrimeric G proteins (a G protein activator). CCDC88A has been implicated in the regulation of continuous and dynamic remodeling of cell morphology, including lamellipodia formation, cell migration, and invasion [25,26,27,28].

Despite the established roles of NLGN2 in synaptic organization [7,8,9,10], it remains unclear whether and how NLGN2 influences neuronal morphogenesis. Additionally, it remains unclear whether NLGN2 exerts its effects through CCDC88A and G protein-ELMO signaling. Addressing this question is important for understanding how NLGN2 dysfunction contributes to the cellular abnormalities associated with NDDs. In the present study, we investigated the molecular mechanisms underlying excessive neuronal process formation induced by *NLGN2* depletion using the N1E-115 cell line, a well-established model that undergoes neurite formation during differentiation [29,30], and primary cortical neurons [31,32]. We demonstrate that the clustered regularly interspaced short palindromic repeat (CRISPR)/Cas13 [33,34]-mediated knocking down of *NLGN2* activates CCDC88A, resulting in elevated Rac1 activity and exaggerated process elongation. The downstream signaling controlling this elongation is mediated through the canonical ELMO-DOCK signaling cascade [35,36]. Our findings identify a previously unrecognized signaling mechanism linking *NLGN2* deficiency to abnormal neuronal morphogenesis and provide insight into a potential recovery strategy for correcting developmental defects associated with NLGN2 dysfunction, at least at the molecular and cellular levels.

## 2. Results

### 2.1. Knockdown of NLGN2 Leads to Activation of CCDC88A

NLGN2 is a single transmembrane protein involved not only in postsynaptic formation and maintenance but also in molecular mechanisms that can contribute to neuronal morphogenesis during early developmental stages [11,12,13,14]. Therefore, we investigated whether knockdown of *NLGN2* is associated with the activity of CCDC88A, another regulator of cell morphology [25,26].

**Figure 1:**
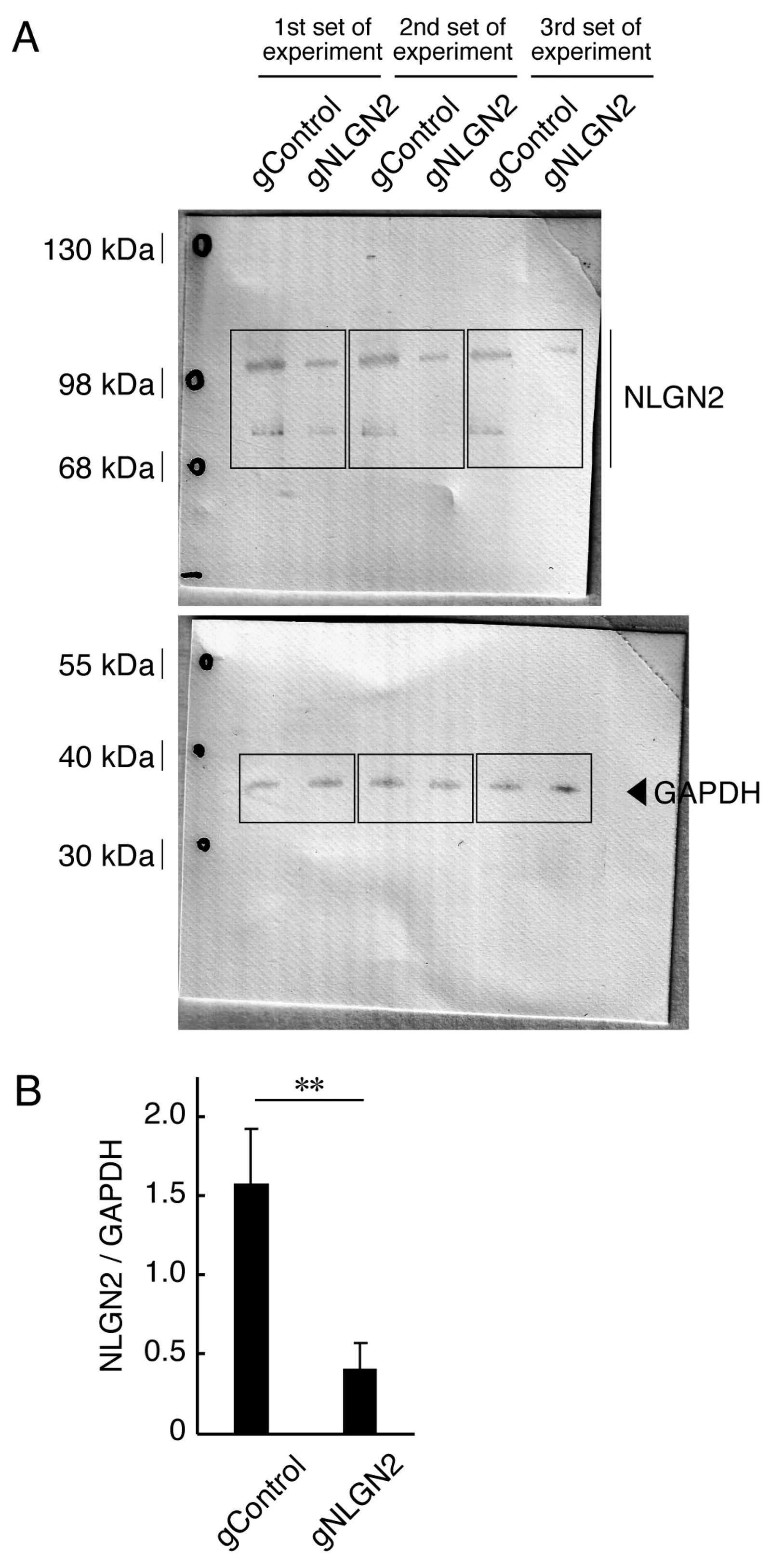
Knockdown of NLGN2 protein in cells (for gNLGN2-105). (**A**,**B**) N1E-115 cells were transfected with Cas13 together with either a control gRNA or a gRNA targeting *NLGN2* (targeting nucleotide position 105 onwards). Lysates were immunoblotted using antibodies against NLGN2 or the internal control glyceraldehyde-3-phosphate dehydrogenase (GAPDH). Full-size gel images are shown, and the regions containing the immunoreactive bands are outlined by square boxes. NLGN2 protein levels were quantified by normalizing the intensity of each marker to that of GAPDH and were subjected to statistical analysis (** *p* < 0.01; *n* = 3 independent blots).

To address this hypothesis, we first knocked down *NLGN2* in N1E-115 cells. Transfection of plasmids encoding CRISPR/Cas13 [33,34]-fitted gRNA (gNLGN2-105) specific for *NLGN2*, but not control gRNA, together with Cas13, resulted in decreased NLGN2 protein expression by approximately 75% (Figure 1A,B). Given its effective knockdown efficiency, this gRNA was basically used for all subsequent experiments.

**Figure 2:**
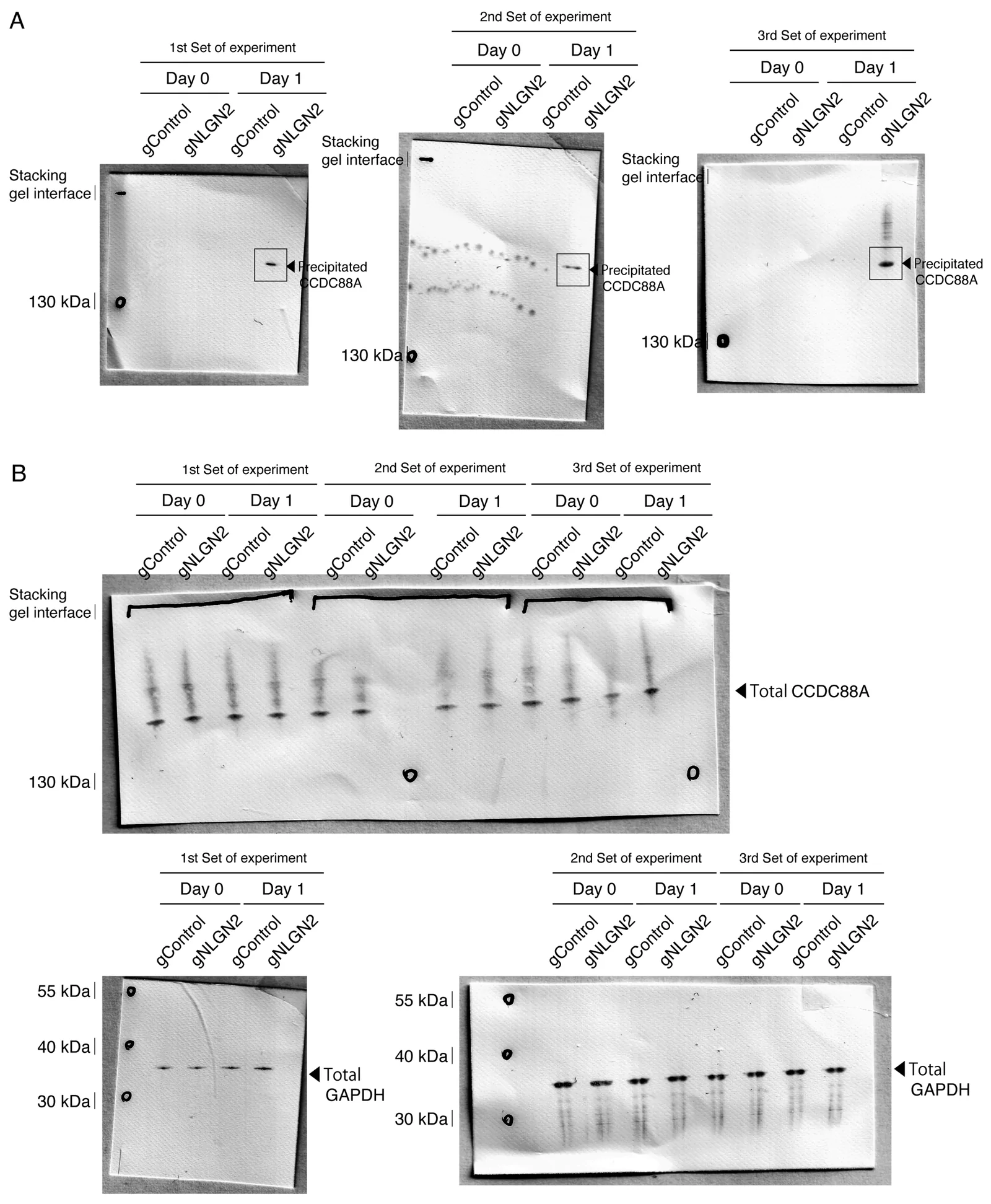
Affinity-precipitation of CCDC88A following depletion of NLGN2. (**A**,**B**) N1E-115 cells were transfected with Cas13 together with either a control gRNA or a gRNA targeting *NLGN2* (targeting nucleotide position 105 onwards). Lysates at days 0 and 1 following the induction of differentiation were used for a CCDC88A affinity-precipitation assay with guanine-nucleotide-free GNAI2 to detect active, precipitated CCDC88A (specifically indicated by square boxes). Lysates were immunoblotted using antibodies against NLGN2 or the internal control GAPDH. Full-size gel images are shown (three sets of experiments).

Utilizing this system, we examined the effect of *NLGN2* knockdown on CCDC88A, a non-receptor guanine nucleotide exchange factor for G protein. We performed an affinity-precipitation assay for CCDC88A protein under *NLGN2* or control gRNA transfection conditions. Since the activated form of guanine nucleotide exchange factors generally binds to the guanine-nucleotide-free form of the effector protein [15,21,22], active CCDC88A was affinity-precipitated using recombinant GST-tagged, guanine-nucleotide-free GNAI2 protein, a G protein subunit. As expected, knockdown of NLGN2, but not transfection with control gRNA, resulted in specific detection of affinity-precipitated CCDC88A (Figure 2A,B), suggesting that CCDC88A is activated following depletion of NLGN2. It is thought that the finding is consistent with our proposed role for CCDC88A under *NLGN2* loss-of-function conditions.

We also knocked down NLGN2 in N1E-115 cells using a second non-overlapping gRNA (gNLGN2-351) targeting a distinct nucleotide region of *NLGN2*. Transfection of plasmids encoding Cas13 together with a CRISPR/Cas13-fitted gRNA specific for NLGN2 or a control gRNA significantly reduced NLGN2 protein expression by approximately 75% compared with the control gRNA (Figure S1). Importantly, this second gRNA produced a comparable reduction in NLGN2 expression (Figure 1A,B) and was also used in process elongation experiments following NLGN2 knockdown (Figure S2).

### 2.2. Knockdown of NLGN2 Exhibited Excessive Process Elongation Phenotypes

Next, we knocked down *NLGN2* and allowed N1E-115 cells to differentiate. *NLGN2* knockdown, but not the control knockdown, significantly promoted excessive process elongation, accompanied by increased expression of the neuronal cell differentiation markers growth-associated protein 43 (GAP43) and β3 tubulin (Figure S3). In contrast, expression levels of the internal control protein actin remained unchanged following differentiation.

### 2.3. Signaling Through CCDC88A Mediates the Excessive Process Elongation Phenotype Induced by NLGN2 Knockdown

**Figure 3:**
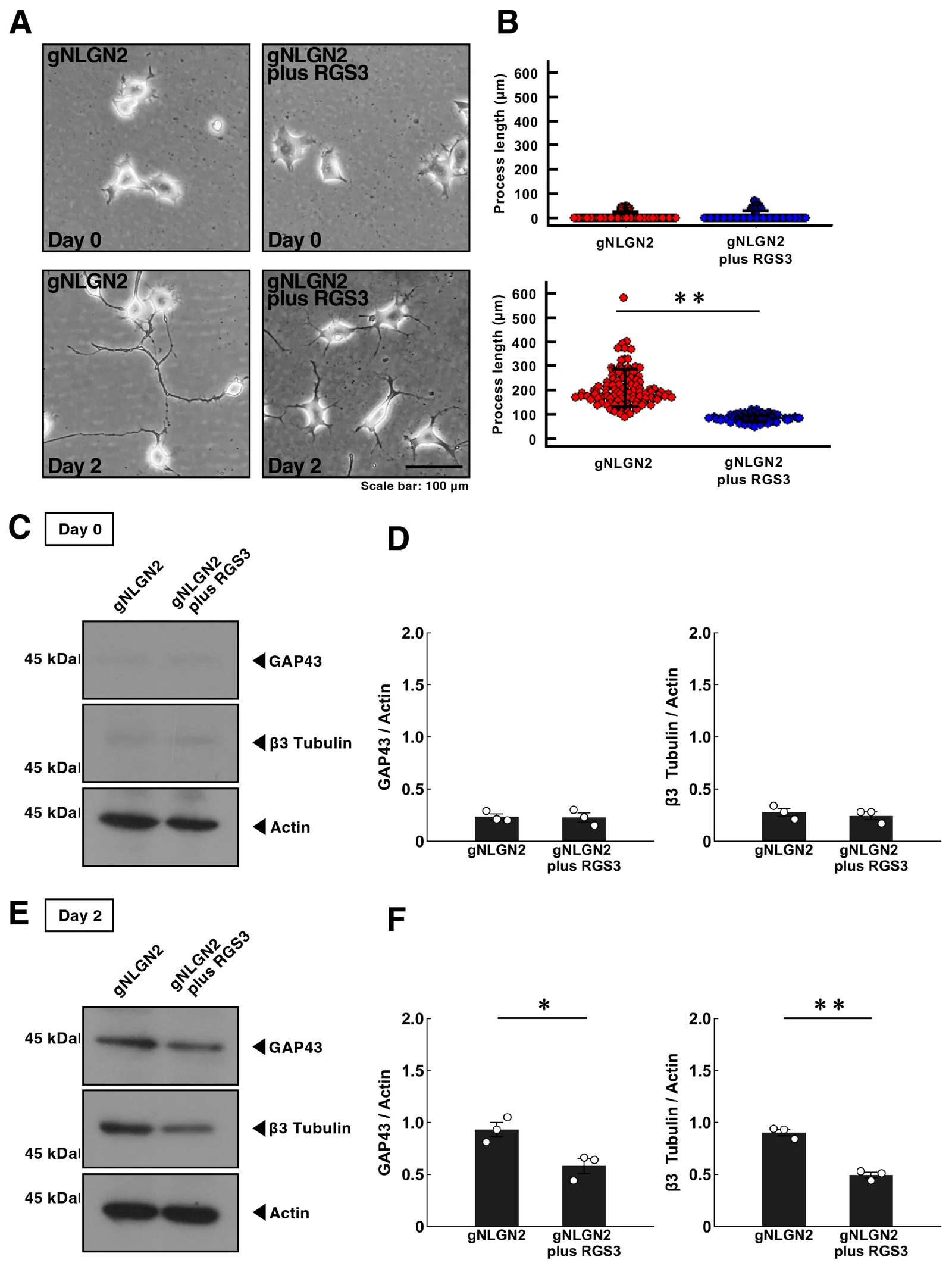
RGS3 (heterotrimeric G protein inhibitor) recovers the excessive process elongation phenotype. (**A**,**B**) N1E-115 cells harboring Cas13 and an *NLGN2*-targeting gRNA were transfected with either an expression plasmid encoding the RGS domain of RGS3 (hereafter termed RGS3) or the corresponding empty vector. Following differentiation induction, cells were cultured for 0 or 2 days. Representative micrographs are shown. The longest process in each cell was measured and quantified (** *p* < 0.01; *n* = 100 cells). (**C**–**F**) Cell lysates collected at 0 or 2 days after differentiation induction were subjected to SDS-PAGE and immunoblotted using antibodies against the neuronal differentiation markers GAP43 and β3-tubulin, with actin serving as a loading control (left panels). Protein levels (right panels) were quantified by normalizing the intensity of each marker to that of actin as the internal control protein and were subjected to statistical analysis (** *p* < 0.01 and * *p* < 0.05; *n* = 3 independent blots).

Since knockdown of NLGN2 stimulated CCDC88A, we next asked whether CCDC88A participates in excessive cell morphological changes. To investigate whether G proteins acting downstream of CCDC88A contribute to this phenotype, we transfected cells with a plasmid encoding the regulator of the G protein signaling (RGS) domain of RGS3, a GTPase-activating protein (negative regulator) for multiple classes of G proteins [25,26]. Transfection of the RGS domain effectively suppressed the excessive process elongation induced by *NLGN2* knockdown, restoring process length toward control levels (Figure 3A,B and Figure S2). Furthermore, expression levels of GAP43 and β3 tubulin were concomitantly decreased (Figure 3C–F), supporting RGS3 attenuation of the excessive process elongation by *NLGN2* knockdown.

**Figure 4:**
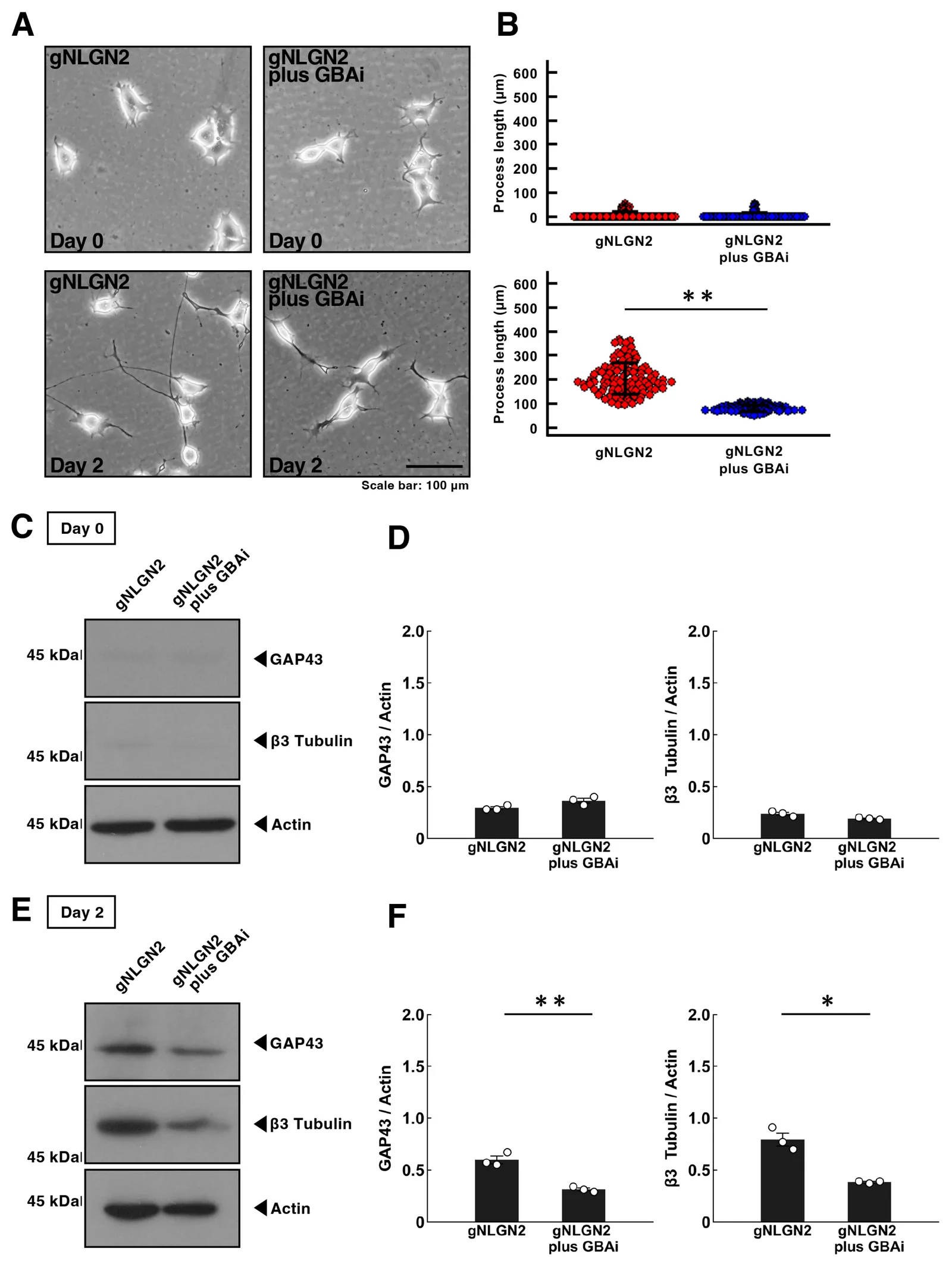
GBAi (CCDC88A protein inhibitor) recovers the excessive process elongation phenotype. (**A**,**B**) Plasmids encoding GBAi or the corresponding control vector were transfected into N1E-115 cells harboring gRNA targeting *NLGN2* and Cas13. After induction of differentiation, cells were cultured for 0 or 2 days. Representative images are shown. The length of the longest process was measured and depicted statistically (** *p* < 0.01; *n* = 100 cells). (**C**–**F**) Lysates from cells at day 0 or day 2 post-differentiation were immunoblotted with antibodies against neuronal differentiation marker proteins GAP43 and β3 tubulin, or the internal control marker protein actin (left panels). Protein levels (right panels) were quantified by normalizing the intensity of each marker to that of actin as the internal control protein and statistically analyzed (** *p* < 0.01 and * *p* < 0.05; *n* = 3 independent blots).

We used GBAi, an inhibitor of the interaction between CCDC88A and GNAI family G proteins [23,24]. Transfection of GBAi, but not the control vector, decreased the excessive process elongation induced by *NLGN2* knockdown (Figure 4A,B and Figure S2). Consistently, expression levels of GAP43 and β3 tubulin were also reduced by GBAi (Figure 4C–F).

Taken together, these findings implicate CCDC88A in the cellular alterations observed following *NLGN2* knockdown, including abnormal process elongation. Similar morphological changes were also observed in primary cortical neurons (Figure S4).

### 2.4. G Protein-Binding Domain of ELMO1 Suppresses the Excessive Process Elongation Phenotype Induced by NLGN2 Knockdown

**Figure 5:**
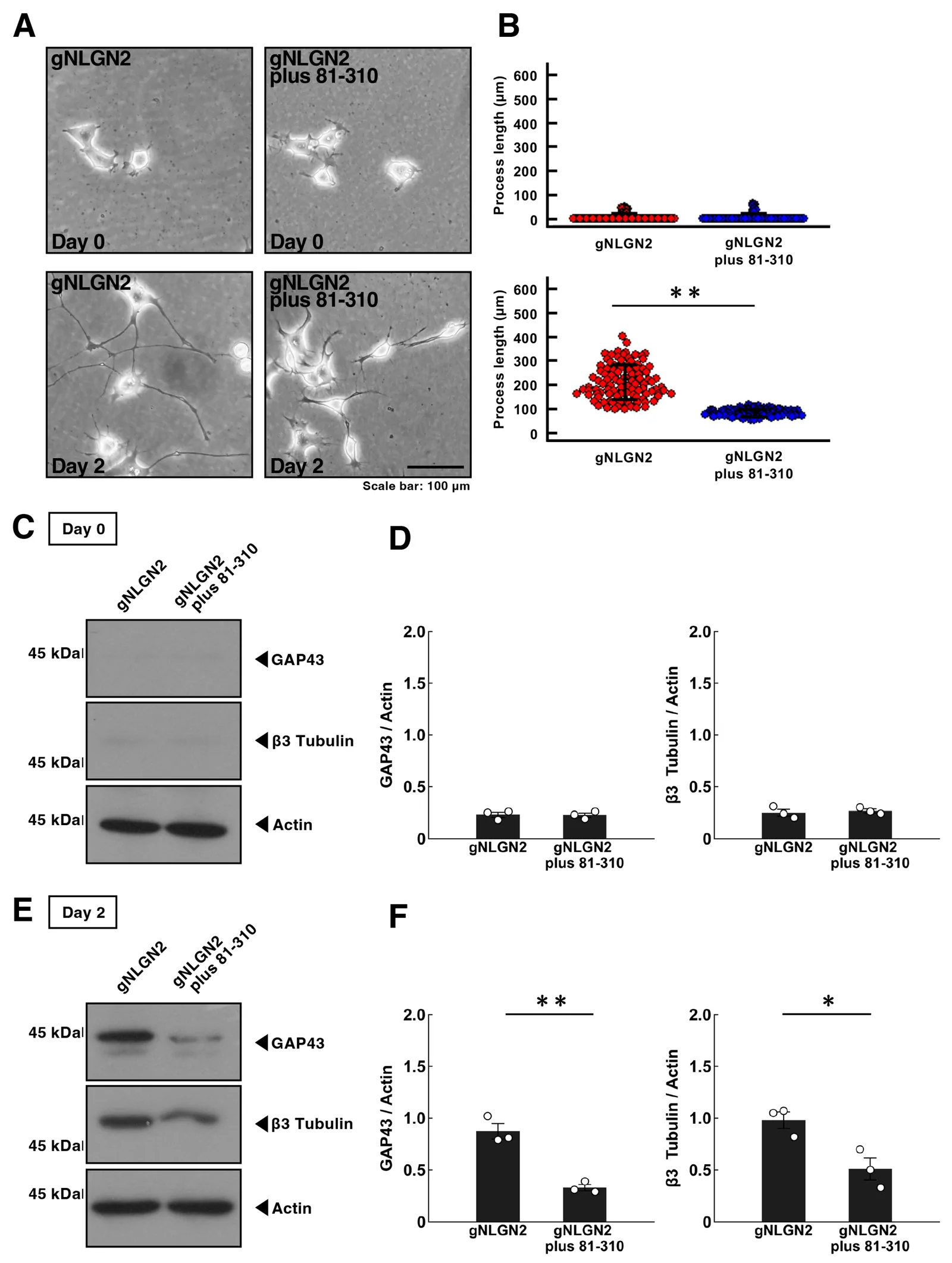
Amino acids 81–310 (G protein-binding domain) of ELMO1 recovers the excessive process elongation phenotype. (**A**,**B**) Plasmids encoding amino acids 81–310 of ELMO1 (hereafter referred to as 81–310) or the corresponding control vector were transfected into N1E-115 cells harboring gRNA targeting *NLGN2* and Cas13. After induction of differentiation, cells were cultured for 0 or 2 days. Representative images are shown. The length of the longest process was measured and depicted statistically (** *p* < 0.01; *n* = 100 cells). (**C**–**F**) Lysates from cells at day 0 or day 2 post-differentiation were immunoblotted with antibodies against neuronal differentiation marker proteins GAP43 and β3 tubulin, or the internal control marker protein actin (left panels). Protein levels (right panels) were quantified by normalizing the intensity of each marker to that of actin as the internal control protein and statistically analyzed (** *p* < 0.01 and * *p* < 0.05; *n* = 3 independent blots).

Given the role of the ELMO-DOCK signaling complex in promoting neurite outgrowth [35,36], we asked whether the abnormal process elongation induced by *NLGN2* depletion was mediated through ELMO-DOCK signaling. We therefore investigated whether the G protein-binding domain of ELMO1 (amino acids 81–310), which couples G proteins to DOCK-A/B family-mediated signaling [21,22], could attenuate the excessive process elongation induced by *NLGN2* knockdown. Transfection of the ELMO1 G protein-binding domain suppressed both excessive process elongation (Figure 5A,B and Figure S2) and the elevated expression of neuronal marker proteins (Figure 5C–F) in *NLGN2*-knockdown cells. Similar results were observed in primary cortical neurons (Figure S4).

### 2.5. These Interventions Normalized the Elevated Rac1 Activity Induced by NLGN2 Knockdown

**Figure 6:**
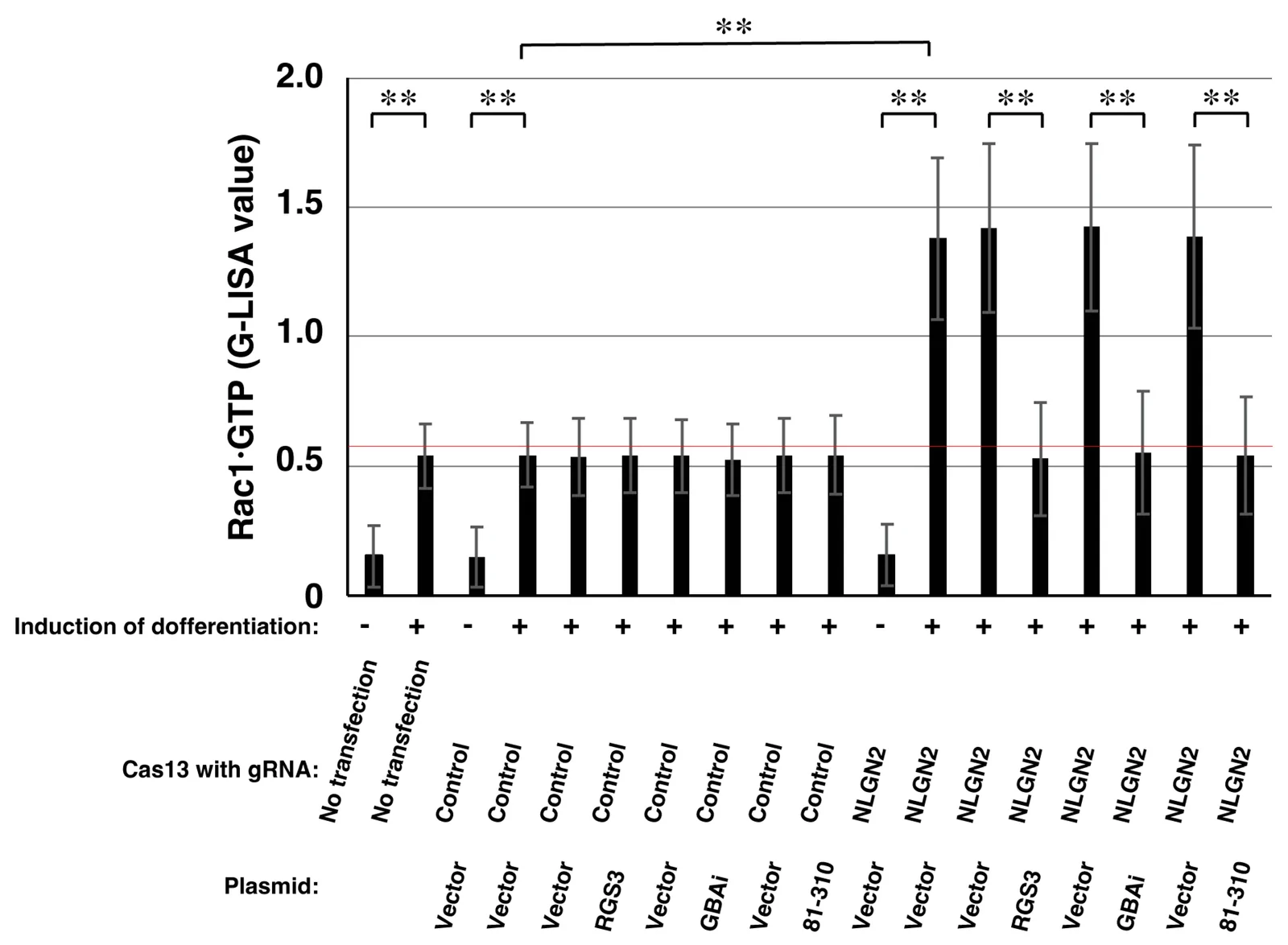
Each G protein-related construct recovers excessive Rac1 activation. N1E-115 cells stably harboring gRNA targeting *NLGN2* or control gRNA and Cas13 were transfected with plasmids encoding the respective constructs (RGS domain of RGS3 [RGS3], GBAi, or amino acids 81–310 of ELMO1 [81–310]) or its empty vector (vector) and cultured for 2 days with (+) or without (-) differentiation induction. Cell lysates were analyzed using a G-LISA GTPase kit to assay the amounts of GTP-bound Rac1 (** *p* < 0.01; *n* = 6). A red and gray reference lines were added to indicate the approximate position of the Rac1 activity level in control differentiated cells and positions at 0.5 intervals, respectively.

Finally, we examined whether the inhibitory molecules used above could normalize the activity of Rac1, a master regulator of cell morphogenesis [19,20], in N1E-115 cells. Plasmids encoding the RGS domain of RGS3, GBAi, or the G protein-binding domain were transfected into control or *NLGN2*-knockdown cells, and Rac1 activity (Rac1·GTP levels) was assessed. All three inhibitory constructs restored Rac1 activity in *NLGN2*-knockdown cells to levels comparable to those observed in control cells (Figure 6).

These findings indicate that the inhibitory molecules attenuate the elevated Rac1 activity induced by *NLGN2* knockdown, supporting the involvement of a CCDC88A–G protein–ELMO signaling axis in regulating neuronal morphogenesis.

## 3. Discussion

The dysregulation of neuronal process elongation, manifesting as either hypertrophic or restricted growth, is widely recognized as an underlying etiology in various neurological and neurodevelopmental disorders [1,2,3,4,5,6]. Emerging evidence suggests that failure in proper morphogenesis during neurodevelopment can trigger NDDs, implicating such morphological abnormalities, especially enhanced neuronal network formation, as a core pathogenic mechanism in conditions like ASD, ID, and ADHD [37,38].

To explore the pathophysiological relevance of these findings, we utilized a CRISPR/Cas13-mediated knockdown to mimic the decreased expression or deficiency of NLGN2 protein, which is often implicated in NDDs [11,12,13,14]. Human genetic studies have identified various de novo nonsense or loss-of-function mutations in the *NLGN2* gene among patients with ASD and ID, many of which are predicted to severely compromise protein stability or cell-surface trafficking [11,12,13,14]. While the clinical phenotypes of these individuals have been predominantly attributed to structural and functional deficits at inhibitory synapses [7,8,9,10], our observations in both N1E-115 cells and primary cortical neurons introduce a possible novel dimension to NLGN2-associated pathogenesis. The excessive process elongation triggered by *NLGN2* depletion may mirror the hypertrophic neuronal growth and aberrant network hyperconnectivity that are increasingly recognized as hallmark structural endophenotypes in the brains of individuals with NDDs [7,8,9,10,11,12,13,14].

In the present study, we investigated the molecular cascades driving these morphological aberrations induced by *NLGN2* knockdown. Although NLGN2 has been extensively studied as a postsynaptic organizer of inhibitory synapses [11,12,13,14], our findings suggest that NLGN2 also acts as a negative regulator of neuronal process elongation. Knockdown of *NLGN2* resulted in activation of Rac1, leading to exaggerated process elongation. These findings support our proposal that NLGN2 contributes to maintaining appropriate levels of morphogenetic signaling in neuronal cells as well as non-neuronal cells [39]. Consistent with this notion, evidence from studies of NLGN1 suggests that neuroligins may participate in the modulation of neuronal cell morphological changes [40,41,42], although the underlying mechanisms remain to be fully elucidated. Interestingly, in contrast to NLGN2, NLGN1 has been reported to promote neurite outgrowth in certain contexts [43,44].

Our data identify CCDC88A as a signaling component activated under potential *NLGN2* loss-of-function conditions. Since CCDC88A acts as a non-receptor guanine nucleotide exchange factor for heterotrimeric G proteins and regulates cytoskeletal remodeling [25,26], its activation provides a plausible mechanistic link between *NLGN2* depletion and excessive process extension [25,26,45]. Several lines of evidence support the involvement of the CCDC88A-G protein signaling axis. First, knockdown of *NLGN2* increased the activated form of CCDC88A. Second, inhibition of the CCDC88A-G protein interaction by GBAi suppressed the excessive process elongation phenotype. Third, inhibition of G protein signaling by the RGS domain of RGS3 produced a similar effect. Collectively, these findings suggest that signaling through G proteins is required for the morphological abnormalities induced by *NLGN2* depletion.

Evidence suggests that proteins of both the NLGN family, including NLGN2, and the NRXN family are involved in regulating neuronal cell morphogenesis during early development [11,12,13,14]. Furthermore, in adult stages, NLGN2, a postsynaptic cell adhesion molecule at inhibitory synapses, directly interacts trans-synaptically with presynaptic NRXN2 to form an adhesion complex [9,10]. Given that both molecules share the same intracellular signaling pathway in our experimental systems [46], this common cascade may coordinately regulate presynaptic differentiation via NRXN2 and postsynaptic organization via NLGN2. Such bidirectional alignment within the same signaling mechanism likely ensures the precise morphological matching of synapses, particularly inhibitory ones.

It is noteworthy that ELMO signaling acts downstream of CCDC88 and G proteins. ELMO proteins act together with DOCK family proteins to activate Rac1, promoting cell morphological changes in many types of cells [21,22]. Rac1 is a central regulator of actin cytoskeletal rearrangement and is also involved in the regulation of neurite formation [19,20]. Expression of the G protein-binding domain of ELMO1 effectively attenuated both excessive process elongation and Rac1 activation in *NLGN2*-knockdown cells. These observations are consistent with previous reports demonstrating coupling between G proteins and ELMO proteins [23,24] and suggest that aberrant activation of the ELMO-Rac1 axis contributes to the morphological phenotype observed following *NLGN2* depletion. While appropriate Rac1 activation generally promotes differentiation and neurite extension [15,16,17,18,19,20], excessive Rac1 activity can lead to abnormal process morphology.

## 4. Materials and Methods

### 4.1. Key Materials

Key materials used in this study are listed in Table 1.

### 4.2. Cell Line Culture and Differentiation

Mouse neuronal cell line N1E-115 (kindly provided by the Human Health Science Foundation Cell Bank, Tokyo, Japan) was cultured on cell and tissue culture dishes (Thermo Fisher Scientific, Waltham, MA, USA) in high-glucose Dulbecco’s modified Eagle medium (DMEM; Nacalai Tesque, Kyoto, Japan, or Fujifilm Wako Pure Chemical, Osaka, Japan) supplemented with 10% heat-inactivated fetal bovine serum (FBS; Thermo Fisher Scientific, Waltham, MA, USA) and a penicillin–streptomycin mixture (Nacalai Tesque, Kyoto, Japan). Cells were maintained at 37 °C in a humidified incubator containing 5% CO_2_, as described previously [35,36].

To generate *NLGN2*-knockdown cells, *NLGN2* gRNA cloned into pSINsi-mU6 (Takara Bio, Kyoto, Japan) was co-transfected with a Cas13 expression plasmid (Addgene, Watertown, MA, USA). Transfected cells were selected with G418 (0.2 mg/mL; Nacalai Tesque) for 2 weeks according to the manufacturer’s G418 selection protocols. Established clones were subsequently maintained under standard culture conditions and used without cryopreservation.

Unless otherwise indicated, differentiation was induced by culturing cells in DMEM supplemented with 1% FBS and penicillin–streptomycin for 0 or 2 days [35,36]. Following differentiation, the length of the longest cellular process was quantified in a blinded manner using the NeuronJ add-in (version 1.4.3; NIH, Bethesda, MD, USA) implemented in ImageJ (version Java 8; NIH). Dot plots were generated using R (version 4.6.0; R Foundation for Statistical Computing, Vienna, Austria).

In these experiments, less than 5% of adherent cells were positive for trypan blue (Nacalai Tesque) under these conditions.

### 4.3. Primary Cell Culture and Axon Elongation

Animals were handled in accordance with the ARRIVE guidelines (https://arriveguidelines.org/ accessed on 1 April 2026), and all studies were conducted according to committee-approved protocols, as described in the Ethics Statement Section. Mice were euthanized by chemical anesthesia using an intraperitoneal injection of a mixture of 0.3 mg/kg medetomidine, 4 mg/kg midazolam, and 5 mg/kg butorphanol.

Primary cortical neurons were isolated from the cerebral cortices of embryonic day 16 to 17 C57BL/6JJcl mice as previously described [31,32]. Briefly, cortical tissues were enzymatically dissociated with papain (100 U/mL) in Leibovitz’s L-15 medium at 37 °C for 15 min and mechanically triturated to generate a single-cell suspension. Cells were then plated onto poly-L-lysine-coated culture dishes (Nacalai Tesque) at a density of 3 to 5 × 10^5^ cells/cm^2^ [31,32]. Neurons were cultured in Neurobasal medium supplemented with 2% B27 (Thermo Fisher Scientific), 1% GlutaMAX (Thermo Fisher Scientific), and 0.1 mg/mL gentamicin. Cells were maintained at 37 °C in a humidified atmosphere containing 5% CO_2_. The culture medium was completely replaced 24 h after plating, and thereafter, half of the medium was refreshed every 2 to 3 days. After 7 to 14 days in vitro, primary cortical neurons were dissociated using 0.05% trypsin containing 0.53 mM EDTA and preserved in liquid nitrogen until further use.

For process elongation assays, cells were replated onto culture dishes and cultured for several days to permit the extension of cellular processes. Following induction of differentiation, the length of the longest processes (axons) was measured in a blinded manner using the NeuronJ (https://imagescience.org/meijering/software/neuronj accessed on 1 April 2026) add-in implemented in ImageJ (https://imagej.net/ij/ accessed on 1 April 2026). Data are presented as dot plots.

In all experiments, fewer than 5% of attached cells were stained with trypan blue under these conditions.

### 4.4. Plasmid Transfection

Cells were transfected with the indicated expression plasmids in pcDNA3.1(+) or pCMV5 using ScreenFect A (Fujifilm Wako Chemicals) according to the manufacturer’s instructions. For Cas13-mediated gene knockdown, cells were co-transfected with a Cas13 expression vector and a Pol III promoter-driven gRNA construct [35,36]. Transfections were performed in serum- and antibiotic-free DMEM. Serum was added 4 h after transfection, and the medium was replaced 24 h later. Cells were subsequently differentiated for 0 or 48 h before being subjected to the indicated cell biological and biochemical analyses unless otherwise indicated.

Under these conditions, fewer than 5% of attached cells were estimated to be trypan blue-positive in each experiment.

### 4.5. Polyacrylamide Gel Electrophoresis and Immunoblotting

Cells were lysed in lysis buffer (50 mM HEPES-NaOH, pH 7.5, 150 mM NaCl, 3 mM MgCl_2_, 1 mM dithiothreitol [DTT], 1 mM phenylmethanesulfonyl fluoride [PMSF], 0.02 mM leupeptin, 1 mM EDTA, 1 mM Na_3_VO_4_, 10 mM NaF, and 0.5% NP-40) [35,36]. Protein samples were prepared by mixing collected supernatants with Laemmli sample buffer (Nacalai Tesque) followed by heat denaturation. Samples were separated by sodium dodecyl sulfate–polyacrylamide gel electrophoresis (SDS-PAGE) on precast gels (Nacalai Tesque) and transferred onto polyvinylidene fluoride (PVDF) membranes (Merck, Darmstadt, Germany). After blocking with Blocking One (Nacalai Tesque), membranes were probed with the indicated primary antibodies and horseradish peroxidase (HRP)-conjugated secondary antibodies. Protein bands were visualized using X-ray film (Fujifilm, Tokyo, Japan) or TMB solution (Nacalai Tesque). Images were digitized using a CanoScan LiDE4000 scanner and CanoScan software (version 2025; Canon). Representative immunoblots from at least three independent experiments are shown. Densitometric analyses were performed using ImageJ software, and values were expressed relative to control samples (internal control actin proteins) where appropriate. All full-sized gel images saved on computers are placed in the supplemental figures (Figures S5–S8, corresponding to Figure 3, Figure 4 and Figure 5 and Figure S3).

### 4.6. Immunoprecipitation and Affinity Precipitation

Cells were lysed in extraction buffer (50 mM HEPES-NaOH, pH 7.5, 150 mM NaCl, 10 mM MgCl_2_, 1 mM DTT, 1 mM PMSF, 0.02 mM leupeptin, 1 mM EDTA, 1 mM Na_3_VO_4_, 10 mM NaF, and 0.5% NP-40) in the presence or absence of 0.1 mM GDP or GTP [35,36]. Centrifuged cell supernatants were incubated for 30 min with antibody-conjugated Protein G-resin (Cytiva, Marlborough, MA, USA) or glutathione-resin preloaded with GST-tagged guanine nucleotide–free G protein subunit alpha-i2 (GNAI2, Proteintech, Rosemont, IL, USA). The protein-resin complexes were recovered by low-speed centrifugation and washed with lysis buffer. After resuspension in sample buffer and heat denaturation, the samples were subjected to SDS-PAGE followed by immunoblotting to detect precipitated active proteins as well as total proteins.

### 4.7. Assay for Active GTP-Bound Rac1

Cells were lysed using the lysis buffer supplied with the G-LISA Rac1 Activation Assay Kit (Cytoskeleton, Inc., Denver, CO, USA) according to the manufacturer’s instructions. Equal amounts of cell lysates were added to Rac1·GTP affinity capture wells and incubated as recommended. After washing, bound Rac1·GTP was detected using an Rac1-specific primary antibody followed by an HRP-conjugated secondary antibody. The signal was developed using a colorimetric HRP substrate and quantified according to the manufacturer’s protocol.

### 4.8. Statistical Analyses

Values are expressed as the mean ± standard deviation (SD) from independent experiments. Comparisons between two groups were conducted using unpaired Student’s *t*-tests implemented in Microsoft Excel (ver. 2023; Microsoft, Redmond, WA, USA). For multiple group comparisons, one-way analysis of variance (ANOVA) followed by Tukey’s honestly significant difference (HSD) post hoc test was performed using the StatPlus Excel add-in (ver. 2021; AnalystSoft, Alexandria, VA, USA). A *p*-value of fewer than 0.05 was considered statistically significant.

### 4.9. Ethics Statement

All animal experiments were conducted in accordance with protocols approved by the Tokyo University of Pharmacy and Life Sciences Animal Care and Use Committee (No. L26-20, approved 1 April 2026). Gene experiments were conducted according to protocols approved by the Tokyo University of Pharmacy and Life Sciences Gene Committee (No. LSR3-01, approved 1 April 2026).

## 5. Conclusions and Perspectives

Here we report that *NLGN2* knockdown results in elevated Rac1 activity, whereas inhibition of CCDC88A, G proteins, or ELMO signaling normalized Rac1 activation. Concomitantly, these interventions rescued the excessive process elongation phenotype. These findings suggest that dysregulated Rac1 signaling is a major downstream effector responsible for the morphological abnormalities induced by *NLGN2* deficiency. Although N1E-115 cells provide a useful model for mechanistic analyses, confirmation in primary neurons suggests that the CCDC88A–G protein–ELMO axis may also operate in developing neurons. Additionally, the observation that depletion of a cell adhesion molecule enhances process elongation suggests that similar signaling mechanisms may operate downstream of other NDD-associated cell adhesion molecules. These findings may deepen our understanding of the molecular and cellular mechanisms underlying NLGN2-related NDDs. Also, they may help guide the development of potential therapeutic strategies to correct these abnormalities.

## 6. Limitations of This Study

This study has several limitations that should be considered when interpreting the findings.

First, our conclusions are based on in vitro systems, including N1E-115 cells and primary cortical neurons. Although these models are useful for dissecting molecular mechanisms, they do not fully reflect the complexity of in vivo neuronal tissue formation. In particular, it remains unclear whether the excessive neuronal process elongation observed following *NLGN2* knockdown directly leads to abnormal neuronal connectivity or behavioral phenotypes. Validation using *NLGN2*-related disease model animals will therefore be necessary to determine the in vivo relevance of the proposed mechanism.

Second, the present study does not include human-derived cellular models. Given the heterogeneity and species-specific aspects of NDDs, it is important to examine whether similar phenotypes are observed in human systems. Future studies using induced pluripotent stem cell (iPSC)-derived neurons will help establish the translational relevance of our findings.

Third, although we have identified CCDC88A as a key mediator of excessive process elongation, the detailed molecular mechanism linking NLGN2 to CCDC88A remains unresolved. Specifically, how NLGN2 regulates CCDC88A activity has not been fully elucidated. Future studies focusing on the role of the interactions between NLGN2 and CCDC88A, as well as the identification of potential intermediate signaling components, will be crucial for unraveling the complete precise pathway.

## Figures and Tables

**Table 1:** Key materials used in this study.

Reagents or Materials	Companies or Sources	Cat. Nos.	Concentrations Used
Key antibodies			
Ant-growth-associated protein 43 (GAP43)	Santa Cruz Biotechnology (Santa Cruz, CA, USA)	sc-17790	Immunoblotting (IB), 1:5000
Anti-beta 3 type tubulin (TUBB3)	Santa Cruz Biotechnology	sc-51670	IB, 1:500
Anti-actin (also called pan-beta type actin)	MBL (Tokyo, Japan)	M177-3	IB, 1:5000
Anti-glyceraldehyde-3-phosphate dehydrogenase (GAPDH)	Santa Cruz Biotechnology	sc-32233	IB, 1:5000
Anti-coiled-coil domain containing 88A (CCDC88A, also called Girdin)	Thermo Fisher Scientific (Waltham, MA, USA)	MA5-47719	IB, 1:250; and immunoprecipitation (IP), 1 microgram for 1 mg of total proteins
Anti-neuroligin 2 (NLGN2)	Thermo Fisher Scientific	PA5-72822	IB, 1:250
Anti-rabbit IgG (goat) pre-absorbed peroxidase-conjugate secondary antibody	Nacalai Tesque (Koyo, Japan)	21858-24	IB, 1:10,000
Anti-mouse IgG (goat) pre-absorbed peroxidase-conjugate secondary antibody	Nacalai Tesque	21860-61	IB, 1:10,000
Recombinant DNAs			
pCMV-SV40ori	A control construct was generated using Addgene Cat. No. 72035 as a template	generated in this study	1.25 mg of DNA per 6 cm dish
NLS-RfxCasRx-NLS (mammalian cell expression plasmid for Cas13)	Addgene (Watertown, MA, USA)	109049	1.25 microgram of DNA per 6 cm dish
pSINsi-mU6	A control construct was generated using GenScript Cat. No. J4052QWTG0 as a template	generated in this study	1.25 microgram of DNA per 6 cm dish
pSINsi-mU6-gRNA for luciferase (targeting 105th nucleotide of open reading frame), which has no significant homology to other mouse transcripts	GenScript (Piscataway, NJ, USA)	generated in this study	1.25 microgram of DNA per 6 cm dish
pSINsi-mU6-gRNA for NLGN2 (targeting 105th nucleotide of open reading frame), which has no significant homology to other mouse transcripts	GenScript	J4052QWTG0	1.25 microgram of DNA per 6 cm dish
pSINsi-mU6-gRNA for NLGN2 (targeting 351st nucleotide of open reading frame), which has no significant homology to other mouse transcripts	GenScript	J6261816G0-2	1.25 microgram of DNA per 6 cm dish
pcDNA3.1(+)-N-eGFP-human RGS3-RGS domain (amino acids 776-917), whose domain acts as GTPase-activating protein (GAP)	GenScript	J761U166G0	1.25 microgram of DNA per 6 cm dish
pLic-myc-GBAi, which acts as inhibitor of Girdin signaling	Addgene	171753	1.25 microgram of DNA per 6 cm dish
pcDNA3.1(+)-N-eGFP-human Elmo1 (amino acids 81-310), which interacts with heterotrimeric G protein subunits	GenScript	J336RUADG0-2	1.25 microgram of DNA per 6 cm dish
pEGFP-C1 (mammalian cell GFP expression plasmid)	Takara Bio (Kyoto, Japan; discontinued product)	Not applicable	1.25 microgram of DNA per 6 cm dish

## Data Availability

The datasets used and/or analyzed during the current study are available from the corresponding author on reasonable request.

## Supplementary Materials

The following supporting information can be downloaded at https://www.mdpi.com/article/10.3390/ijms27177612/s1.
