# Supplementary material for: Modeling NDD-Associated NLGN2 Depletion Using CRISPR/Cas13 Reveals Exaggerated Process Elongation Mediated by the CCDC88A-G Protein–ELMO Axis
Source: Int J Mol Sci. 2026 Aug 25;27(17):7612. doi: 10.3390/ijms27177612 (PMC13566496; doi:10.3390/ijms27177612)
Supplement: Supplementary file 1 [file ijms-27-07612-s001.zip › 02.2. Supplemental figure legends.pdf]

Supplemental figure legends

Figure S1. Knockdown of NLGN2 protein in cells (for gNLGN2-351). (A, B) N1E-115 cells were transfected with Cas13 together with either a control gRNA or a gRNA targeting *NLGN2* (targeting nucleotide position 351 onwards). Lysates were immunoblotted using antibodies against NLGN2 or the internal control GAPDH. Full-size gel images are shown, and the regions containing the immunoreactive bands are outlined by square boxes. NLGN2 protein levels were quantified by normalizing the intensity of each marker to that of GAPDH and were subjected to statistical analysis (** $p < 0.01$; n = 3 independent blots).

Figure S2. Effects of control or *NLGN2* knockdown on N1E-115 cells (for gNLGN2-351). (A-C) N1E-115 cells harboring gRNA targeting *NLGN2* (targeting nucleotide position 351 onwards) or control gRNA and Cas13 were transfected with plasmids encoding the respective constructs and cultured for 2 days following the induction of differentiation. The length of the longest process was measured and depicted statistically (** $p < 0.01$; n = 100 cells).

Figure S3. Effects of control or *NLGN2* knockdown on N1E-115 cells. (A, B) N1E-115 cells stably harboring gRNA targeting *NLGN2* or control gRNA and Cas13 were cultured for the indicated days following the induction of differentiation. Representative images are shown. The length of the longest process was measured and depicted statistically (** $p < 0.01$; n = 100 cells). (C-F) Lysates from cells at the indicated day following the

induction of differentiation were immunoblotted using antibodies against neuronal differentiation marker proteins GAP43 and β3 tubulin, or the internal control marker protein actin (left panels). The band intensity of each marker protein (right panels) was normalized to that of actin as the internal control and statistically analyzed (* $p < 0.05$; n = 3 independent blots).

Figure S4. Effects of transfection of G protein signaling components in primary cortical neurons. (A, B) Primary cortical neurons isolated from mouse cortical tissue were transfected with plasmids encoding the respective constructs and pEGFP-C1 for morphological visualization, together with gRNA targeting *NLGN2* gRNA and Cas13, and cultured for the indicated days. The length of an axonal process was measured and depicted statistically (** $p < 0.01$; n = 200 cells). Representative images are shown.

Figure S5. Computer-saved full-size gel images for Figure 3.

Figure S6. Computer-saved full-size gel images for Figure 4.

Figure S7. Computer-saved full-size gel images for Figure 5.

Figure S8. Computer-saved full-size gel images for Figure S3.
